# Diabetes, glucose tolerance, and the risk of sudden cardiac death

**DOI:** 10.1186/s12872-016-0231-5

**Published:** 2016-02-24

**Authors:** Antti Eranti, Tuomas Kerola, Aapo L. Aro, Jani T. Tikkanen, Harri A. Rissanen, Olli Anttonen, M. Juhani Junttila, Paul Knekt, Heikki V. Huikuri

**Affiliations:** Department of Internal Medicine, Päijät-Häme Central Hospital, Keskussairaalankatu 7, 15850 Lahti, Finland; Division of Cardiology, Heart and Lung Center, Helsinki University Central Hospital, Helsinki, Finland; Medical Research Center Oulu, Oulu University Hospital and University of Oulu, Oulu, Finland; Department of Health, Functional Capacity, and Welfare, National Institute for Health and Welfare, Helsinki, Finland

**Keywords:** Death, sudden, cardiac, Diabetes mellitus, Diabetic cardiomyopathies, Prediabetic state, Prospective studies

## Abstract

**Background:**

Diabetes predisposes to sudden cardiac death (SCD). However, it is uncertain whether greater proportion of cardiac deaths are sudden among diabetes patients than other subjects. It is also unclear whether the risk of SCD is pronounced already early in the course of the disease. The relationship of impaired glucose tolerance (IGT) and SCD is scarcely documented.

**Methods:**

A general population cohort of 10594 middle-aged subjects (mean age 44 years, 52.6 % male, follow-up duration 35–41 years) was divided into diabetes patients (*n* = 82), subjects with IGT (*n* = 3806, plasma glucose ≥9.58 mmol/l in one-hour glucose tolerance test), and controls (*n* = 6706).

**Results:**

Diabetes patients had an increased risk of SCD after adjustment confounders (hazard ratio 2.62, 95 % confidence interval 1.46–4.70, p = 0.001) but risk for non-sudden cardiac death was similarly increased and the proportion of SCD of cardiac deaths was not increased. The SCD risk persisted after exclusion of subjects with baseline cardiac disease or non-fatal cardiac events during the follow-up. Subjects with IGT were at increased risk for SCD (univariate hazard ratio 1.51; 95 % confidence interval 1.31–1.74; *p* < 0.001) and also for non-sudden cardiac deaths and non-fatal cardiac events but adjustments for other risk factors attenuated these effects.

**Conclusions:**

Diabetes was associated with increased risk of SCD but also the risk of non-sudden cardiac death was similarly increased. The proportion of cardiac deaths being sudden in subjects with diabetes was not increased. The higher SCD risk in diabetes patients was independent of known cardiac disease at baseline or occurrence of non-fatal cardiac event during the follow-up.

## Background

Sudden cardiac death (SCD) is estimated to account for 50 % of deaths from cardiovascular causes and about half of these deaths occur in subjects who are not previously diagnosed with heart disease [1, 2]. Coronary artery disease is the underlying cause in 80 % of SCDs, consequently, risk factors for coronary artery disease also predispose to SCD [1]. Diabetes is a well-established risk factor for coronary artery disease [3]. Diabetes also predisposes patients to heart failure – an important risk factor for SCD – independent of other risk factors of heart failure [2–4]. In addition to coronary artery disease and heart failure, cardiac autonomic neuropathy (CAN), a late complication of diabetes, might explain the increased incidence of SCD among diabetes patients. Increased prevalence of silent myocardial ischemia and QT interval prolongation have been documented among diabetes patients with CAN and this at least partly explains the observed three-fold increase in mortality and higher proportion of deaths attributed to SCD among subjects with CAN [5]. Interestingly, changes in measures of autonomic function have also been documented in subjects with glucose levels in the prediabetic range [6].

Expectedly, diabetes has been shown to be associated with approximately two-fold risk of SCD [7]. However, epidemiologic evidence suggests that diabetes might not be specifically associated with the risk of SCD as it is generally thought that also the risk of non-sudden cardiac death increases similarly among diabetes patients although opposite evidence exists as well [8, 9]. The timing of the SCD risk in the course of diabetes has not been comprehensively studied but it warrants attention as coronary artery disease generally develops early in the course of diabetes but heart failure and CAN are late complications [3]. Also prediabetic dysglycemic states, such as impaired fasting glucose (IFG) and impaired glucose tolerance (IGT) are associated with increased cardiovascular mortality but little is known of the association of IGT and SCD [10]. In this work we studied the risks of sudden and non-sudden cardiac deaths associated with IGT and diabetes in subjects with and without heart disease. We also tested whether QT interval lengthening and acceleration of heart rate, electrocardiographic markers of CAN, would separate diabetes patients dying suddenly from cardiac causes.

## Material and methods

### Study population

The study population consists of 10957 men and women aged 30–59 years who took part in the Coronary Heart Disease Study of the Finnish Mobile Clinic (FMC) Health Examination Survey between 1966 and 1972. The survey was carried out in 12 localities in Finland and the sample represented well the Finnish middle-aged population. The overall participation rate was 89.6 % (total 12310 invited subjects). The study rationale and procedures performed at the baseline examinations have been described previously [11]. At baseline a standard 12-lead electrocardiogram (ECG) was recorded and blood pressure, body mass index (BMI), and serum cholesterol were measured. The subjects also completed a questionnaire regarding their health habits, known diseases, medication, smoking habits, and symptoms of cardiovascular disease. Subjects who had not been diagnosed with diabetes had an oral glucose tolerance test (OGTT) but for practical reasons a venous blood sample was drawn only once at 1 h after the glucose load. The sample was centrifuged immediately and separated plasma samples were frozen for analyses 1–3 weeks later. Plasma glucose concentration was measured by an autoanalyzer modification (Technicon Auto-Analyzer Methodology N2- b) of the ferricyanide reduction method. The glucose load as a 20 % solution was intended to be 40 g/m^2^ body surface area but for practical reasons fixed doses of 60, 75, or 90 g were used the 75 g dose being the most frequently administered [11]. The type of diabetes was not specifically documented during the data collection but it is likely that a major part of the subjects using insulin had type 1 diabetes and subjects treated with tablet medication or diet had type 2 diabetes. The ECGs were interpreted by nine trained readers and coded according to the revised Minnesota code (MC) [11, 12]. We excluded subjects with missing or unreadable ECGs (*n* = 53), subjects in whom the OGTT was not performed for reasons other than known diabetes (*n* = 121), subjects in whom the assessment of QT interval corrected for heart rate according to Bazett’s formula (QTc) was unreliable (*n* = 51, 16 of these due to atrial fibrillation), subjects with missing blood pressure (*n* = 128), subjects with 2^nd^ or 3^rd^ degree atrioventricular block (*n* = 1), or Wolff-Parkinson-White-pattern (*n* = 4) and also rest of the subjects with atrial fibrillation or flutter (*n* = 5), and thus we were left with 10594 subjects for data-analysis. The FMC survey precedes current legislation on ethics in medical research. All participants were fully informed about the study, they participated in the study voluntarily and the use of the information for medical research was explained to them. Agreeing to participate in the baseline health examination was taken to indicate informed consent. The follow-up study using record linkage to the Care Register for Health Care (THL/924/6.02.00/2011) was approved by the Ethical Committee of the National Institute for Health and Welfare on June 8, 2011. Statistics Finland approved linkage to Causes of Death Register (TK-53–588–12). These registries decide on providing data for research purposes based on an application procedure.

### Follow-Up

From the baseline examination the subjects were followed for 35–41 years until the end of 2007. Less than 2 % of the subjects were lost to follow-up, but also for the majority of this group the survival status could still be determined. The mortality data were obtained from the Causes of Death Register maintained by Statistics Finland. The death certificates were obtained for each deceased. All deaths from cardiac causes were reviewed by two experienced cardiologists (O.A. and H.V.H.) by the use of hospital records and necropsy reports, if available, to identify sudden deaths from arrhythmia based on the definitions presented in the Cardiac Arrest Pilot Study [13], as described by our group previously [14]. Briefly, SCD was defined as a spontaneous cessation of respiration and circulation with loss of consciousness either instantaneously or preceded by symptoms attributable to arrhythmia or myocardial ischemia in the absence of heart failure. Also unwitnessed deaths without evidence of another cause were classified as SCD. In addition, episodes of coronary heart disease and congestive heart failure requiring hospitalization were obtained from the Care Register for Health Care (HILMO) maintained by The National Institute for Health and Welfare which records nationwide data on all inpatient episodes in Finland. The accuracy of these registers has proven to be good [15].

### Statistical analyses

Due to the lack of a standard cutoff point for plasma glucose concentration in 1-h OGTT in diagnosing IGT we used a Receiver operating characteristics (ROC) analysis to determine the 1-h glucose value providing maximal sensitivity plus specificity in discriminating subjects whose cause of death was classified as SCD from other subjects and used this value as the cutoff for IGT. The differences in characteristics of subjects with normal glucose tolerance, IGT and diabetes were compared using ANOVA for continuous variables and Chi-Square test for categorical variables. The Chi-Square test was used to compare proportions of cardiac deaths and non-cardiac deaths and SCD and non-sudden cardiac deaths. Our primary outcome was SCD, but analyses were also made with non-sudden cardiac death, and a composite of hospitalization for coronary heart disease and congestive heart failure as the end-point. The hazard ratios (HRs) and their 95 % confidence intervals (95 % CIs) were calculated using the Cox proportional hazards model. The covariates were selected based on previous evidence of an association with cardiovascular mortality. Age, BMI, systolic blood pressure, cholesterol, QTc, and heart rate were used as continuous variables and sex, smoking status, cardiac disease in the baseline, electrocardiographic signs of myocardial infarction, and electrocardiographic signs of coronary artery disease were used as binary variables. A subject was defined as having cardiac disease in the baseline if a “yes” answer was present in the baseline questionnaire in at least one of the following questions: Do you have or have you had 1) valvular disease, 2) congestive heart failure, 3) angina pectoris, 4) myocardial infarction, or 5) congenital heart disease? Electrocardiographic signs of myocardial infarction were defined by the presence of definitely pathological Q-waves (MC 1–1) or Q-waves in the presence of T wave inversions (MC 1–2 and 5–1 or 5–2). Electrocardiographic signs of coronary artery disease were defined as the presence of minor Q-waves (MC 1–2 or 1–3), ST-segment depressions (MC 4–1, 4–2, or 4–3), T-wave inversions (MC 5–1 or 5–2), or ventricular conduction defects (MC 7–1, 7–2, or 7–4). The follow-up time was defined as the number of days from the baseline examination to the event of interest, death or the end of follow-up, whichever came first. The general linear model was used to assess the age- and sex adjusted means of QTc and heart rate among diabetes patients with different causes of death. All p values are two-sided. The statistics analyses were made with the Statistical Package for Social Studies, version 22 (SPSS).

## Results

The ROC analysis for plasma glucose concentration in 1-h OGTT yielded maximum sensitivity plus specificity in discriminating subjects whose cause of death was classified as SCD at 9.58 mmol/l (172.5 mg/dl). Using this value as the cutoff for IGT, 3806 subjects (35.9 %) had IGT, 82 subjects (0.8 %) had diabetes, and 6706 subjects (63.3 % of all subjects) had normal glucose tolerance.

### Baseline characteristics of subjects

The baseline characteristics of subjects are presented in Table 1. Subjects with IGT and diabetes were generally older, had higher cholesterol, body mass index, blood pressure, heart rate and QTc, and they had more often electrocardiographic signs of coronary artery disease than subjects with normal glucose tolerance. When the baseline characteristics of males and females were compared in the groups of normal, IGT, and diabetes patients, QTc and heart rate were lower in men in all groups. Systolic and diastolic blood pressures were higher among women with diabetes than men with diabetes. Smoking and left ventricular hypertrophy were more prevalent in all groups of men than in women (data not shown). The treatments used among diabetes patients were insulin (*n* = 24), sulphonylureas (*n* = 23) or biguanides (*n* = 8) or both (*n* = 9), and diet (*n* = 18).

**Table 1:** Baseline characteristics of subjects

	Normal (*n* = 6706)	IGT (*n* = 3806)	Diabetes (*n* = 82)	*P*
Male, %	53.3	51.4	57.3	0.142
Age, years	42.9 (8.2, 42.7–43.1)	45.7 (8.5, 45.4–45.9)	50.1 (7.6, 48.5–51.8)	<0.001
BMI, kg/m^2^	25.6 (3.6, 25.5–25.7)	26.3 (4.1, 26.2–26.5)	28.5 (5.3, 27.3–29.6)	<0.001
Systolic blood pressure, mmHg	134.8 (19.8, 134.3–135.2)	144.6 (22.7, 143.8–145.3)	148.4 (25.6, 142.8–154.0)	<0.001
Diastolic blood pressure, mmHg	80.6 (11.8, 80.4–80.9)	84.7 (13.0, 84.3–85.2)	87.9 (13.3, 84.9–90.8)	<0.001
Cholesterol, mmol/l	6.4 (1.3, 6.4–6.5)	6.6 (1.4, 6.5–6.6)	6.9 (1.5, 6.6–7.2)	<0.001
Smoker, %	36.0	30.6	30.5	<0.001
Cardiac disease, %	6.9	9.6	23.2	<0.001
Heart rate, bpm	73.6 (14.1, 73.2–73.9)	78.8 (16.5, 78.3–79.3)	79.3 (15.8, 75.8–82.7)	<0.001
QRS duration, ms	86.9 (8.4, 86.7–87.1)	87.1 (8.6, 86.8–87.3)	88.5 (11.9, 85.9–91.1)	0.163
QTc, ms	404.6 (26.9, 403.9–405.2)	414.6 (27.5, 413.7–415.4)	415.4 (31.2, 408.5–422.2)	<0.001
Left ventricular hypertrophy^a^, %	30.5	33.2	25.6	0.008
Electrocardiographic signs of myocardial infarction, %	0.4	0.7	4.9	<0.001
Electrocardiographic signs of coronary artery disease, %	8.5	11.9	23.2	<0.001

^a^Assessed with Sokolow-Lyon electrocardiographic criterion

### Mortality and non-fatal cardiac events

During the follow-up 3503 (52.2 %) subjects with normal glucose tolerance, 2363 (62.1 %) subjects with IGT and 80 (97.6 %) subjects with diabetes died. Mortality was higher among men than among women (63.9 % vs 47.4 %, respectively). The proportions of cardiac and non-cardiac deaths, and sudden and non-sudden cardiac deaths are presented in Table 2. A high percentage of cardiac deaths was observed among males with diabetes, among whom 56.5 % of deaths were cardiac. For females the proportion of cardiac deaths was the same in all groups. The proportions of SCD and non-sudden cardiac deaths were similar among subjects with IGT and diabetes compared to normal subjects. During the follow-up 31.6 % of normal subjects, 34.7 % of subjects with IGT and 52.4 % of diabetes patients were hospitalized for coronary heart disease and 14.9 % of normal subjects, 16.8 % of subjects with IGT and 41.5 % of diabetes patients were hospitalized for congestive heart failure.

**Table 2:** Proportions of cardiac and non-cardiac deaths and sudden and non-sudden cardiac deaths by diabetes status

Causes of death	Normal (*n* = 3504)	IGT (*n* = 2364)	Diabetes (80)	*p*
Non-cardiac	2370 (67.6 %)	1558 (65.9 %)	45 (56.3 %)	0.050
Cardiac	1134 (32.4 %)	806 (34.1 %)	35 (43.8 %)	
Suddenness of cardiac deaths	Normal (*n* = 1134)	IGT (*n* = 806)	Diabetes (*n* = 35)	*p*
Non-arrhythmic	697 (61.5 %)	470 (58.3 %)	23 (65.7 %)	0.302
Arrhythmic	437 (38.5 %)	336 (41.7 %)	12 (34.3 %)	

### Roles of IGT and diabetes as risk factors for SCD and other cardiac events

The roles of IGT and diabetes as risk factors for SCD, non-sudden cardiac death and non-fatal cardiac events were assessed in Cox proportional hazard models. The results of these models are presented in Table 3. IGT was associated with increased risk of SCD, non-sudden cardiac death and non-fatal cardiac events only in the univariate models. Diabetes was strongly associated with SCD and similarly with non-sudden cardiac deaths and non-fatal cardiac events even after a comprehensive multivariate adjustment. Adjustment for electrocardiographic abnormalities had minor impact on the HRs. These models were also conducted separately for males and females giving similar results with the exception of the risk of SCD among females with diabetes in which the risk estimate was uncertain because only one female with diabetes died of SCD.

**Table 3:** The HRs for sudden and non-sudden cardiac death and for non-fatal cardiac events

Sudden cardiac death	Normal (*n* = 6706)	IGT (*n* = 3806)	*P*	Diabetes (*n* = 82)	*P*
No. of events	437	336		12	
Unadjusted HR	1 (reference)	1.51 (1.31–1.74)	<0.001	5.52 (3.10–9.82)	<0.001
Model 1^a^	1 (reference)	1.16 (1.00–1.35)	0.045	2.62 (1.46–4.70)	0.001
Model 2^b^	1 (reference)	1.14 (0.98–1.32)	0.088	2.40 (1.34–4.33)	0.003
Non-sudden cardiac death	Normal (*n* = 6706)	IGT (*n* = 3806)	*p*	Diabetes (*n* = 82)	*p*
No. of events	697	470		23	
Unadjusted HR	1 (reference)	1.36 (1.21–1.53)	<0.001	8.43 (5.50–12.81)	<0.001
Model 1^a^	1 (reference)	0.94 (0.83–1.06)	0.321	3.05 (1.99–4.67)	<0.001
Model 2^b^	1 (reference)	0.92 (0.82–1.04)	0.194	2.90 (1.89–4.46)	<0.001
Non-fatal cardiac events	Normal (*n* = 6706)	IGT (*n* = 3806)	*p*	Diabetes (*n* = 82)	*p*
No. of events	2483	1573		56	
Unadjusted HR	1 (reference)	1.27 (1.19–1.35)	<0.001	6.47 (4.95–8.44)	<0.001
Model 1^a^	1 (reference)	0.99 (0.92–1.05)	0.693	3.63 (2.77–4.75)	<0.001
Model 2^b^	1 (reference)	0.99 (0.92–1.05)	0.680	3.50 (2.67–4.59)	<0.001

^a^Model 1: Adjusted for age, sex, BMI, systolic blood pressure, cholesterol, smoking, and baseline cardiac disease

^b^Model 2: Adjusted for age, sex, BMI, systolic blood pressure, cholesterol, smoking, baseline cardiac disease, heart rate, QTc, electrocardiographic signs of myocardial infarction, and electrocardiographic signs of coronary artery disease

### Subgroup analyses

The Cox proportional hazards models with SCD and non-sudden cardiac death as end-point were also conducted among subjects who were not hospitalized due to coronary heart disease or congestive heart failure during the follow-up and who were free of cardiac disease at baseline. There were 4031, 2088, and 24 subjects and 206, 146, and 4 SCDs in groups of normal, IGT and diabetes patients, respectively. In these models IGT and diabetes predicted SCD univariately (IGT HR 1.50, 95 % CI 1.22–1.86, *p* < 0.001 and diabetes HR 8.40, 95 % CI 3.11–22.65, *p* < 0.001). In the multivariate models (variables as in Table 2 excluding baseline cardiac disease) only diabetes predicted SCD significantly, the hazard ratios being 4.21 (95 % CI 1.55–11.40, *p* = 0.005) and 3.52 (95 % CI 1.28–9.67, *p* = 0.015) for models 1 and 2, respectively. The results were virtually similar when non-sudden cardiac death was the end-point in corresponding models.

To assess the risk of SCD, non-sudden cardiac death and non-fatal cardiac events in patients with probable type 2 diabetes (diabetes patients not treated with insulin), the models presented in Table 3 were re-conducted after exclusion of diabetes patients treated with insulin. The results remained virtually the same in these models. To lessen the impact of new diabetes cases presenting during the follow-up, we also conducted separate models in which the follow-up time was truncated to 10 years. The multivariate-adjusted hazard ratios for SCD and non-fatal cardiac events among diabetes patients were similar to those in models with complete follow-up but the multivariate adjusted hazard ratio for non-sudden cardiac death was smaller and no statistical significance was reached in these models. However, there were only 4 SCDs and 3 non-sudden cardiac deaths among diabetes patients during the first 10 years of follow-up which led to wide confidence intervals.

### QTc, heart rate and causes of death among diabetes patients

The general linear model was used to calculate age- and sex-adjusted mean QTc and heart rate, markers of CAN, among diabetes patients and compared the means according to the cause of death. These results are presented in Table 4. The means for QTc were slightly longer and heart rate was slightly faster among diabetes patients who had died of cardiac causes but no statistical significance was reached in these analyses.

**Table 4:** Mean QTc and heart rate among diabetes patients with different causes of death

	*n*	Mean QTc, ms (SD, 95 % CI)	*p* ^a^	Mean heart rate, bpm (SD, 95 % CI)	*p* ^a^
Alive or non-cardiac death	47	412 (31, 402–421)		78 (16, 73–82)	
Non-sudden cardiac death	23	419 (32, 406–432)	0.360	81 (12, 75–87)	0.441
Sudden cardiac death	12	423 (33, 404–442)	0.290	81 (21, 72–90)	0.565

^a^The *p* values test the difference of group mean compared to the mean of group alive/non-cardiac death

## Discussion

In this study we assessed the risk of SCD and other cardiac events associated with diabetes and IGT. Special emphasis was on whether the risk of SCD would be pronounced over the risk of non-sudden cardiac death among diabetes patients and the timing of the SCD risk associated with diabetes.

We expectedly observed that diabetes was associated with increased risk of SCD and also with increased risk of non-sudden cardiac death. However, diabetes was not specifically related to the risk of SCD as the proportion of SCD of all cardiac deaths did not increase, and the hazard ratios for SCD and non-sudden cardiac death were largely similar among diabetes patients. The suddenness of cardiac deaths among diabetes patients compared to subjects without diabetes has been under debate but generally diabetes is believed to have similar influence on the risk of SCD and non-sudden cardiac death [8]. This is supported by recent findings from the large population-based Atherosclerosis Risk in Communities study in which both the incidence rate of SCD and non-sudden cardiac death were 4-fold higher and multivariate-adjusted hazard ratios for these end-points were largely similar among diabetes patients. Also the proportions of SCD of all cardiac deaths were virtually the same between diabetes patients and healthy subjects in this study [16]. Some evidence to the contrary also exist as in the Paris Prospective Study diabetes was associated with increased risk of SCD but not for increased risk of non-fatal myocardial infarction [9].

Predictably, the diabetes patients were also at increased risk of hospitalizations for coronary heart disease and congestive heart failure as these conditions are frequent complications of diabetes [3]. Thus, a large portion of diabetes patients had either or both coronary heart disease and congestive heart failure, both important risk factors for SCD, which explains partly the increased incidence of SCDs among the diabetes patients. However, the diabetes patients were at increased risk of SCD and non-sudden cardiac death also when subjects with overt heart disease were excluded from the models suggesting that diabetes increases the risk of these events, independently of diagnosed cardiac disease. This is a significant observation as it would sound reasonable that the increment in SCD risk associated with diabetes is largely mediated through the increased burden of heart failure, severe diffuse coronary atherosclerosis and CAN which are late complications of diabetes [3]. The matter has received only little attention previously but prior reports are in line with the findings of the present study. In previous prospective studies, inclusion or exclusion of subjects with pre-existing vascular disease did not markedly alter the observed risk of SCD [7]. In a large case–control study the point estimates of HR for SCD associated with diabetes were larger in subjects without heart disease but the models were adjusted for slightly different sets of confounders [17]. In one cohort study based on a female population the HR for SCD associated with diabetes was 2.0 in the whole population and 2.2 when subjects with coronary heart disease were excluded but subjects with heart failure were still included in the model [18].

Thus, it seems that the risk of SCD, non-sudden cardiac death, and non-fatal cardiac events increase similarly in diabetes so the risk of SCD is not specifically pronounced. Finding markers identifying subjects specifically at high risk for SCD from those subjects with an overall elevated risk of death from cardiac causes would be beneficial for efforts to reduce SCD burden in the population. Diabetes seems to lack this specificity as do most of the currently available risk markers of SCD [2]. Diabetes also seems to be associated with increased risk of SCD already before the development of overt heart disease. However, in any future studies with larger numbers of subjects with diabetes these issues should be assessed by stratifying for the type and duration of diabetes. In type 2 diabetes coronary atherosclerosis begins to develop already in a prediabetic state in contrast to the other possibly arrhythmogenic complications of diabetes such as heart failure, CAN, and cardiac fibrosis that appear as later manifestations [3]. The presence of microvascular disease is associated with an increased risk of SCD in diabetes patients [17]. It has also been shown that in myocardial infarction patients with diabetes a larger proportion of deaths are sudden compared to myocardial infarction patients without diabetes [19, 20]. It is reasonable to assume that the diabetes patients who already have had a myocardial infarction or who have developed microvascular disease are in an advanced state of diabetes compared to the ones in prospective population based studies. This may explain the differences in findings between population based studies and the studies mentioned above.

In our study IGT predicted SCD and other cardiac events univariately but adjustment for confounders attenuated this effect. IGT is a risk factor for diabetes and part of the metabolic syndrome which is an established risk factor for cardiovascular disease, but it is difficult to assess the individual contribution of IGT as it often presents with other important risk factors such as hypertension, dyslipidemia, obesity, and proinflammatory and prothrombotic states [21]. In a recent systematic review with follow-up times of individual studies ranging from 5 to 21.5 years, the pooled relative risk of fatal and non-fatal cardiac events associated with IGT was 1.20 (95 % CI 1.07–1.34) but the degree of adjustment was limited in many of the included studies [22]. These data, together with the results of the present study suggest that no notable changes in the risk are associated with IGT even in a longer follow-up. The relationship of IGT and SCD risk has been less thoroughly investigated. In the Paris Prospective Study I middle-aged males with IGT were at increased risk for SCD based on analysis of cumulative mortality curves [9]. In a 23-year follow-up of men of Japanese ancestry living on the island of Oahu on Hawaii the risk of SCD gradually increased with worsening glucose tolerance in a 1-h OGTT despite multivariate adjustment [23]. The relative risks of SCD in that study were 1.59 and 2.22 in subjects with OGTT results 8.39–12.44 and ≥12.45 mmol/l, respectively, compared to subjects with normal glucose tolerance [23]. Interestingly, in recent study impaired fasting plasma glucose was shown to be associated with a 1.5-fold risk of SCD after a comprehensive multivariate adjustment in a male population [24]. Thus, also prediabetic hyperglycemic states seem to predict SCDs in addition to other cardiac events but the topic warrants further studies to define their final value in risk stratification.

We did not find significant differences in mean QTc or heart rate in diabetes patients grouped according to their cause of death. However, our sample of subjects with diabetes was small to detect differences in such variables with large between-subject variance. A high resting heart rate is characteristic in patients with CAN and there is a particularly strong association between CAN and QTc lengthening [25, 26]. Previously, in one case–control study consisting of diabetes patients free of cardiac disease at baseline, the subjects in the fourth QT interval quartile were at 3-fold increased risk for SCD compared with the first quartile [27]. However, in that study the mean follow-up time from the ECG to event among SCD victims was 5.1 years whereas in our study among diabetes patients it was 12.8 years which may give an explanation as to why we did not observe differences in QTc and heart rate. It may be that the subjects of the present study developed CAN, QTc lengthening, and higher heart rate only during the follow-up. Anyhow, given the previous encouraging results of the use of QTc and heart rate in SCD risk stratification among diabetes patients this issue should be studied in larger populations of diabetes patients in the future.

The strengths of our study include a large population and a long and comprehensive follow up. The main weakness of our study was that the amount of subjects with diabetes was relatively small. In addition, we were not able to identify subjects with undiagnosed diabetes or IGT in the baseline according to present-day diagnostic criteria. Historically, there were multiple protocols for the OGTT and only minor emphasis was on the 2-h glucose value used today [28]. Therefore, some subjects with diabetes might have been in the IGT group. One could also argue that the cutoff used to classify abnormal OGTT result based on the 1-h glucose was too low as the IGT group was relatively large. However, 1-h glucose cutoff as low as 8.61 mmol/l (155 mg/dl) has been shown to be associated with elevated hsCRP levels, increased carotid intima media thickness, impaired insulin sensitivity, future diabetes and cutoff 8.94 mmol/l (161 mg/dl) has been shown to be associated with cardiovascular deaths [29–31]. We were also unable to identify subjects who developed IGT or diabetes during the follow-up, which is likely to somewhat dilute the hazard ratios observed. Finally, the generalizability of our results to contemporary patient populations may be limited because the prevalence of type II diabetes has been increasing during the last decades in contrast to the relatively large proportion of type I diabetes patients in our cohort.

## Conclusions

The risk of SCD among diabetes patients was high in middle-aged general population, even in the absence of established cardiac disease. However, these patients were also at increased risk for non-sudden cardiac death and the proportion of cardiac deaths being sudden among diabetes patients was not elevated. IGT was associated with increased risk of SCD as well, but its independent contribution as a risk factor warrants further research as adjustment for other confounders attenuated this effect.

## Abbreviations

BMI: body mass index
CAN: cardiac autonomic neuropathy
CI: confidence interval
ECG: electrocardiogram
FMC: Finnish Mobile Clinic
HR: hazard ratio
IFG: impaired fasting glucose
IGT: impaired glucose tolerance (plasma glucose ≥9.58 mmol/l in one-hour OGTT)
MC: Minnesota Code
OGTT: oral glucose tolerance test
QTc: QT interval corrected for heart rate according to Bazett’s formula
SCD: sudden cardiac death
